# Investigation of motility and biofilm formation by intestinal *Campylobacter concisus* strains

**DOI:** 10.1186/1757-4749-4-22

**Published:** 2012-12-14

**Authors:** Peter Lavrencic, Nadeem O Kaakoush, Karina D Huinao, Nupur Kain, Hazel M Mitchell

**Affiliations:** 1 School of Biotechnology and Biomolecular Sciences, The University of New South Wales, Sydney, NSW, 2052, Australia

**Keywords:** *Campylobacter concisus*, Motility, Adherence, Viscous, Mucus, Biofilm

## Abstract

Motility helps many pathogens swim through the highly viscous intestinal mucus. Given the differing outcomes of *Campylobacter concisus* infection, the motility of eight *C*. *concisus* strains isolated from patients with Crohn’s disease (n=3), acute (n=3) and chronic (n=1) gastroenteritis and a healthy control (n=1) were compared. Following growth on solid or liquid media the eight strains formed two groups; however, the type of growth medium did not affect motility. In contrast, following growth in viscous liquid medium seven of the eight strains demonstrated significantly decreased motility. In media of increasing viscosities the motility of *C*. *concisus* UNSWCD had two marked increases at viscosities of 20.0 and 74.7 centipoises. Determination of the ability of UNSWCD to swim through a viscous medium, adhere to and invade intestinal epithelial cells showed that while adherence levels significantly decreased with increasing viscosity, invasion levels did not significantly change. In contrast, adherence to and invasion of UNSWCD to mucus-producing intestinal cells increased upon accumulation of mucus, as did bacterial aggregation. Given this aggregation, we determined the ability of the eight *C*. *concisus* strains to form biofilms, and showed that all strains formed biofilms. In conclusion, the finding that *C*. *concisus* strains could be differentiated into two groups based on their motility may suggest that strains with high motility have an increased ability to swim through the intestinal mucus and reach the epithelial layer.

## Introduction

*Campylobacter jejuni* and *Campylobacter coli* are the most common cause of acute bacterial gastroenteritis world-wide and as a result, they are the most widely studied *Campylobacter* species [1]. In recent years, a number of other *Campylobacter* species, including *Campylobacter concisus*, have emerged as gastrointestinal pathogens [2-4]. For example, *C*. *concisus* has been isolated from faecal samples and colonic intestinal biopsies of patients with both acute and chronic gastroenteritis and Crohn’s disease [5,6]. Although in comparison to *C. jejuni* relatively little is known about *C. concisus*, studies have shown that they share a number of similarities [7]. While both bacteria are spiral shaped and flagellated, *C. jejuni* can have single or bi-polar flagella, whereas *C. concisus* only has a single polar flagellum.

Bacterial flagella are complex, highly refined organelles that allow bacteria to swim through fluids, including viscous environments, and which also play a central role in adhesion to and invasion into host cells [8]. In the well-established pathogen *C. jejuni*, flagellar motility has been reported to be a key pathogenicity factor [9], with early studies showing that *C. jejuni* was capable of colonising the mucus layer and intestinal crypts filled with mucus [10]. Further, the flagellum of *C. jejuni* has been shown to assist in bacterial adhesion to epithelial cells [11]. Scanning electron microscopy (ScEM) studies have shown that *C. concisus* adheres to the intestinal epithelium by wrapping its flagellum around the microvilli of intestinal epithelial cells [5,12]. Although the importance of motility in *C. concisus* has yet to be described, current data would suggest that the flagellum may be an important pathogenicity factor in *C. concisus* infection [5,12].

Bacterial flagella have also been shown to be involved in biofilm formation [13,14]. The ability to form biofilms through the complex interaction of bacteria has been reported to be important for bacterial survival within the human host. A key feature of biofilms in bacterial survival is self-defence. Although bacteria infecting the human body stimulate both an innate and adaptive immune response, neither of these is capable of penetrating and eliminating bacteria within the well-established biofilm [15]. *C. concisus* ATCC 33237, a human gingival isolate, has also been shown to form biofilms *in vitro*[16].

In some bacteria the flagellin protein of the flagellum has been reported to be heavily glycosylated [17], with studies showing that *C. jejuni* and *C. coli* strains contain flagellin glycosylation biosynthesis pathways for the synthesis of two sugars, pseudaminic acid (PA) [17] and legionaminic acid (LA) [13]. Interestingly, studies of *C. jejuni* and *C. coli* have shown that the flagellum mediates auto-agglutination of flagellin glycans [17,18]. It has been postulated that the close proximity of bacteria allows them to interact with adjacent flagella initiating auto-agglutination, aggregation, and the formation of micro-colonies [13]. In a recent study investigating the type of glycosylation pathways in eight strains of *C. concisus*, we showed that seven of the eight *C. concisus* strains contained proteins for the PA pathway, while one strain contained the LA pathway [19]. Thus, it is possible that differences may exist between *C. concisus* strains in their ability to auto-agglutinate their flagellin glycans.

In this study, the motility of eight *C. concisus* strains found to have different virulence potential was determined following growth on three different medium types, and the effects of viscosity on *C. concisus* motility and pathogenesis was elucidated. Moreover, the ability of these eight *C. concisus* strains to form biofilms was assessed.

## Materials and methods

### Bacterial strains and growth conditions

Eight *C. concisus* strains that had been previously isolated from patients with a range of intestinal diseases [5] were included in this study. *C. concisus* strains UNSWCD, UNSW2, UNSW3 (Crohn’s disease), UNSW1 (chronic gastroenteritis), ATCC 51561 (healthy subject), ATCC 51562, UNSWCS and BAA 1457 (acute gastroenteritis) were grown on Horse Blood Agar (HBA) plates [Blood Agar Base No. 2 supplemented with 6% defibrinated horse blood (Oxoid; Heidelberg West, VIC, Australia)], and incubated at 37°C under microaerobic conditions with H_2_ [generated using *Campylobacter* Gas Generating Kits (Cat. #. BR0056A, Oxoid)] for 24 h.

To evaluate motility following growth in liquid medium, the eight *C. concisus* strains were first grown on HBA plates for 24 h, harvested and then transferred to individual 10 ml Brain Heart Infusion (BHI) broths (Oxoid) containing 10% Foetal Bovine Serum (FBS) (Interpath; Heidelberg West, VIC, Australia), and where relevant, a known concentration of carboxy-methyl-cellulose (CMC) (Sigma-Aldrich; Castle Hill, NSW, Australia) that corresponded to a particular viscosity ([20], Additional file 1). The broths were then incubated for 24 h under microaerobic conditions at 37°C.

### Motility assay

*C. concisus* cultures were centrifuged at 3,619 × *g* for 5 min and the cell pellets resuspended in 500 μl of PBS. The OD was then measured at 595 nm, and the OD adjusted to 0.5 (optimisation of the OD value is presented in Additional file 2). To conduct the motility assay, semi-solid serum plates (20 ml) [28 g Brucella broth (BD), 3.5 g Bacteriological Agar No. 1 (Oxoid) and 10% FBS] were inoculated with bacteria (3.8 μl), and then incubated at 37°C under microaerobic conditions for 72 h. After 72 h of incubation, a zone of motility was observed around the inoculation point, which represented the distance that the bacteria had migrated.

### Mammalian cell culture

Two cell lines were used in this study, the human intestinal epithelial cell line Caco-2 (American Type Culture Collection; HTB-37) and the human mucin producing intestinal cell line LS174T (American Type Culture Collection; CL-188).

Caco-2 cells were grown in 10 ml cell culture medium comprised of Minimum Essential Medium (MEM), (Invitrogen; Mulgrave, VIC, Australia) supplemented with 10% FBS, 1 mM sodium pyruvate, 0.1 mM non-essential amino acids, 2.25 mg 1^-1^ sodium bicarbonate and 100 μg ml^-1^ penicillin and streptomycin (Invitrogen) in 25 cm^2^ tissue culture flasks (In Vitro Technologies; Noble Park, VIC, Australia) at 37°C with 5% CO_2_. Cells were seeded at a concentration of 5 × 10^5^ cells ml^-1^ into 24-well plates and kept for 2 days at 37°C with 5% CO_2_ in order to form a confluent monolayer (confirmed visually) for the adherence and invasion assays. Prior to seeding, the wells were coated with 1 ml collagen (0.338 mg ml^-1^) and incubated for 20 min at 37°C with 5% CO_2_.

LS174T cells were grown in 10 ml cell culture medium comprising Roswell Park Memorial Institute (RPMI)-1640 medium (Invitrogen) supplemented with 10% FBS and 100 μg ml^-1^ penicillin and streptomycin in 25 cm^2^ tissue culture flasks at 37°C with 5% CO_2_. Cells were seeded at a concentration of 5 × 10^5^ cells ml^-1^ into 24-well plates and kept for 2 days to form a confluent monolayer (confirmed visually) (0-day time point). The confluent monolayer was incubated at 37°C with 5% CO_2_ for an extra 2 days to allow the development of a mucin layer (confirmed visually) for the adherence and invasion assays (2-day time point). The medium was changed daily until the development of a mucin layer.

### Gentamicin protection (invasion) and adherence assays

Following incubation, the seeded cells in each well were washed twice with 1 ml of the relevant antibiotic-free medium, after which 1 ml of antibiotic-free medium with CMC at a concentration of 0 centipoise (cp), 20 cp or 74 cp (Caco-2 cells only) were aliquoted into the seeded wells. Monolayers were inoculated with *C. concisus* UNSWCD at a multiplicity of infection (MOI) of 200. Infected monolayers were then co-incubated with the bacteria for 6 h at 37°C with 5% CO_2_ to allow adherence and invasion to occur. Invasion and adherence assays were then performed as previously described by Kaakoush *et al.*[5]. Bacterial adherence was calculated by subtracting the internalized bacteria determined using the gentamicin protection assay from the bacterial counts obtained using the adherence assay, and expressed as a relative percentage of inoculated bacteria.

### Western blot analysis

LS174T cells were seeded at 5 × 10^5^ cells ml^-1^ in 24-well plates and incubated at 37°C. After 2 days the cells were lysed and collected using 400 μl radioimmunoprecipitation (RIPA) buffer. A bicinchoninic acid assay was used to determine the protein concentration for each cell lysis sample collected. OD values were measured at 595 nm using the Bio-Rad 8550 Microplate Reader (Bio-Rad; Gladesville, NSW, Australia).

Proteins were then separated on 12% SDS-PAGE gels, and transferred to methanol-treated polyvinylidine difluoride membranes with use of the Trans-blot cell transfer system (Bio-Rad). Membranes were probed in accordance with the Immun-StarWesternC Kit protocol (Bio-Rad). Membranes were immunolabeled with mouse monoclonal antibodies against Mucin-1 (MUC1) (1:200), Mucin-2 (MUC2) (1:200), or β-actin (1:1000) (Santa Cruz Biotechnology Inc.; Santa Cruz, CA, USA). Goat anti-mouse IgG antibodies coupled to HRP (1:2000; Bio-Rad) were used as a secondary antibody. Bands were visualized and quantified using a LAS-3000 (Fujifilm; Brookvale, NSW, Australia).

### *Measurement of Biofilm formation by* Campylobacter concisus

*C. concisus* strains were grown on HBA plates for 24 h, after which the bacteria were harvested, resuspended and the OD measured at 595 nm. The OD was then adjusted with PBS to 0.5. Two aliquots of the harvested bacteria, each of 150 μl, were then evenly distributed over two cover slips in a glass petri dish containing 5 ml BHI supplemented with 10% FBS. The glass petri dish was then incubated at 37°C under microaerobic conditions for 72 h. After 72 h, the medium was carefully removed and the petri dish washed gently with 2 ml of PBS to remove planktonic bacteria. The petri dish was then placed into an 80°C oven for 30 min to heat fix any biofilm formed. A 3 ml aliquot of 0.1% crystal violet (Oxoid) was then added to the petri dish and this was left to stain for 1 h at room temperature. After 1 h, the crystal violet was removed with a pipette and the petri dish was vigorously washed with 2 ml of PBS to remove any excess crystal violet stain. Following this, 2 ml of 95% ethanol was added to the petri dish until all the stain was dissolved. The OD of the dissolved biofilm inside the petri dish was measured at 595 nm. In each experiment a negative control (no bacteria) was included to account for non-specific binding of the stain.

### Statistical analysis

In order to perform statistical analyses on the data, all experiments were repeated a minimum of three times, with each biological replicate consisting of two technical replicates. Analysis of Variance (ANOVA) was conducted using Minitab 15.1.0.0 (Minitab Inc.; State College, PA, USA) for all statistical studies conducted, with the significance level set at *p* < 0.05. The data were checked to ensure that it fitted the assumptions of ANOVA including homogeneity of variance and normal distribution. Tukey’s *post hoc*-multiple comparisons were conducted where significant differences were found. To determine if prior growth on the three different media types affected the motility of the eight *C. concisus* strains, a two-way ANOVA was conducted. In the statistical model, strain and medium were set as fixed factors, and the interaction of these factors were tested. A one-way ANOVA was performed for all other statistical analyses within this study.

## Results and discussion

### *Motility of the eight* Campylobacter concisus *strains*

**Figure 1:**
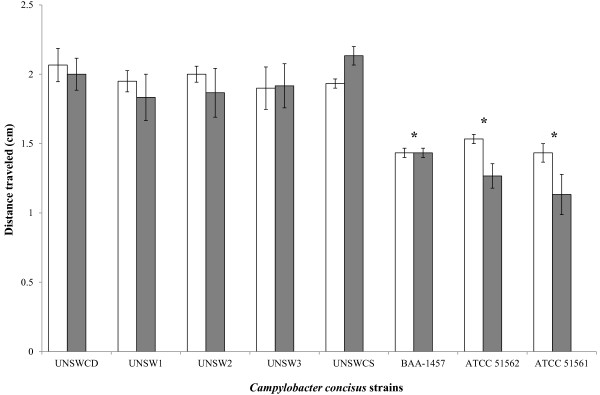
**The motility of *Campylobacter concisus *strains following growth in solid (white) and liquid (gray) media.** Vertical lines associated with histogram bars represent standard error of the mean. * implies *p*<0.05.

The motility of eight strains of *C. concisus* following growth on solid agar and liquid media were measured. All *C. concisus* strains were found to be motile. ANOVA detected significant differences between strains (F_7,73_ = 26.5, *p* < 0.01) (Figure 1). In contrast, the type of growth medium (solid or liquid) had no effect on the motility of any of the eight *C. concisus* strains tested (Figure 1). In relation to their motility, the eight strains formed two distinct groups; UNSWCD, UNSW1, UNSW2, UNSW3 and UNSWCS having higher overall levels of motility than BAA-1457, ATCC 51562 and ATCC 51561 (Figure 1). Interestingly, *C. concisus* UNSWCD, UNSW1, UNSW2 and UNSW3 were originally isolated from patients with chronic gastroenteritis, whereas strains UNSWCS, BAA-1457 and ATCC 51562 were isolated from patients with acute gastroenteritis and strain ATCC 51561 from a healthy individual [5]. While this finding suggests that the level of motility may play a role in disease outcome, the fact that the motility level of UNSWCS, isolated from a patient with acute gastroenteritis, was similar to that of strains isolated from patients with chronic gastroenteritis, would suggest that other pathogenicity factors, may also contribute to virulence. From a biological viewpoint, such differences in motility raise the possibility that strains with high motility may be more capable of swimming through the intestinal mucus and reaching the epithelium, as compared with strains with lower motility which potentially would be lost upon mucus turn-over.

### *Motility of the eight* Campylobacter concisus *strains grown in viscous medium*

The colonic mucus is composed of two semipermeable layers totalling a thickness of 800 μm [21]. The top layer of mucus is less viscous (approximately 20 cp), is much thicker and is normally colonised by commensal bacteria [22]. In comparison, the bottom layer is denser, thinner and much more elastic giving the layer properties that make it impermeable to normal commensal bacteria [22]. Given this, we were interested in determining the effect on bacterial motility following growth in a viscous liquid, as this would mimic to some degree the viscosity encountered in the host, where prior to reaching the intestinal epithelium, the bacteria must move through the intestinal mucus layers that act as a barrier against many pathogens. Due to the lack of homogeneity of the two mucus layers, many *in vitro* studies have used CMC to estimate bacterial motility through an intestinal mucus-like medium [9,23]. The advantages of CMC are that it is non-toxic to tissue culture and bacterial cells, is more chemically defined than purified human mucin, and can be easily manipulated to create the desired viscosity; however, it remains an artificial compound that does not mimic the complex environment within the intestinal tract.

**Figure 2:**
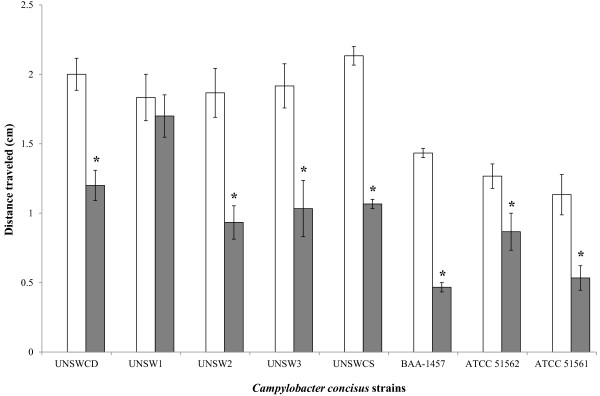
**The motility of *Campylobacter concisus *strains following growth in liquid (white) and 20 cp viscous liquid (gray) medium.** Vertical lines associated with histogram bars represent standard error of the mean. * implies *p*<0.05.

Following growth in a viscous medium (20.0 cp), the motility of the eight *C. concisus* strains showed significant changes (F_2,73_ = 132.0, *p* < 0.0001), and this effect varied between strains (F_14,73_ = 2.0, *p* = 0.03). In seven of the eight *C. concisus* strains motility levels were found to drop significantly as compared with motility levels following growth on solid or liquid media (Figure 2). While the distance travelled for strain UNSW1 slightly decreased following growth in viscous liquid medium, this was not significant (Figure 2). This decrease in motility following growth in viscous medium was unexpected. However, we speculate that this may result from the upregulation of auto-agglutination following the mechanosensing of the viscous environment. This possibility is strengthened by the fact that *C. concisus* strains have been observed to aggregate in large numbers around mucus [5].

### *Motility of* Campylobacter concisus *UNSWCD following culture in varying viscosities*

**Figure 3:**
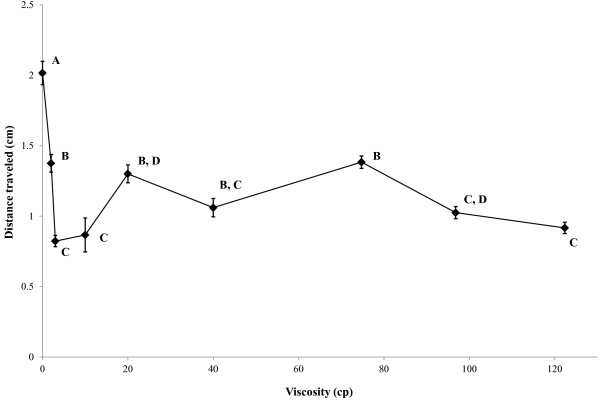
**Motility of *Campylobacter concisus *UNSWCD following growth in liquid medium of varying viscosities.** Vertical lines associated with histogram bars represent the standard error of the mean. Results were analysed using a one-way ANOVA test. In addition, tukey’s *post hoc* multiple comparisons test was conducted to ascertain statistically significant differences. Symbols sharing the same letter, are not statistically different (*p*>0.05) to each other.

To examine more closely the effect of increasing viscosity on *C. concisus* motility, further studies were conducted on *C. concisus* UNSWCD as this strain was shown in the above motility studies to have a high level of motility. Thus, we considered that any differences in motility at differing viscosity levels would be more detectable. Determination of the effect of increasing viscosity levels on *C. concisus* UNSWCD motility showed an initial decrease in the distance travelled (motility) as viscosity increased from 0 cp to 3.0 cp (Figure 3). Interestingly, between the viscosities of 3.0 cp to 20.0 cp, motility levels increased with increasing viscosity, being significantly higher at 20.0 cp than at 3.0 cp (*p* < 0.01) (Figure 3). This viscosity (20.0 cp) represents the approximate viscosity level in the outer mucus layer of the colon [9]. Following this peak in motility, levels fell between 20.0 cp and 40.0 cp, after which a further increase in motility was observed at 74.7 cp (Figure 3). Following 74.7 cp, motility levels dropped as viscosity levels increased. Given that the inner mucus layer of the intestine is thinner and more dense, the finding that *C. concisus* UNSWCD’s motility had a possible second motility peak at higher viscosity (74.7 cp) may suggest that it can adapt its motility to pass through the inner intestinal mucus layer. However, given that the viscosity of the inner intestinal mucus layer remains unknown, no direct comparisons can be made.

Interestingly, two peaks of motility have also been reported in *C. jejuni* which has been postulated to represent an adaptation that assists *C. jejuni* to reach the intestinal epithelial surface [23]. Shigematsu *et al.* suggested that the first increase in velocity was predominantly due to the bacterium’s flagella propelling the motion [23], while the second increase in motility they suggested was due predominantly to the spiral shape of the bacterium. In comparison, this same study showed the motility of the well-established pathogen *Salmonella enterica* Serovar Typhimurium had only one peak of motility at 1.5 cp after which the velocity decreased dramatically as the viscosity increased [23]. Interestingly, although *S.* Typhimurium is flagellated, it does not have a spiral body like *C. jejuni* and *C. concisus*.

### Campylobacter concisus *adherence and invasion into host cells in a viscous environment*

Szymanski *et al.*[9] have previously shown that as viscosity increases from 0 to 141 cp, adherence to and invasion of *C. jejuni* to Caco-2 epithelial cells significantly increases (*p* < 0.05). Thus, we considered it possible that *C. concisus* may show a similar trend. To test this, adherence and invasion assays were performed with the addition of medium at two different viscosity levels following culture of the Caco-2 cells. The two viscosities chosen, 20.0 cp and 74.7 cp, were based on the two peak motilities shown in our studies examining the effect of viscosity on *C. concisus* UNSWCD motility (Figure 3). A control containing medium alone (no CMC) was also examined. The results of the adherence assay showed that *C. concisus* UNSWCD at zero viscosity (0 cp) adhered to Caco-2 cells at a similar level (6.01 ± 0.33%) to that previously reported in a study where centrifugation was used [5]. A finding that would suggest that for UNSWCD centrifugation may not be a necessary step for adherence. At a viscosity of 20.0 cp, *C. concisus* adherence was reduced (4.48 ± 0.53%) and at 74.7 cp it decreased even more (3.48 ± 0.50%) (F_2,21_ = 8.9, *p* = 0.0015). Similar to the adherence assay, the invasion level of *C. concisus* into Caco-2 cells in the absence of centrifugation (0.56 ± 0.08%), was again similar to that reported in a previous study where centrifugation was included (0.47 ± 0.04%) [5]. When the viscosity was increased to 20.0 cp and 74.7 cp, invasion of *C. concisus* into Caco-2 cells remained the same (0.51 ± 0.07% and 0.50 ± 0.09%, respectively) and did not differ from the control (F_2,6_ = 0.17, *p* = 0.84). The observed reduction in *C. concisus* adherence to Caco-2 cells upon exposure to viscous medium is consistent with the hypothesis that *C. concisus* may upregulate auto-agglutination upon mechanosensing the viscous environment.

**Figure 4:**
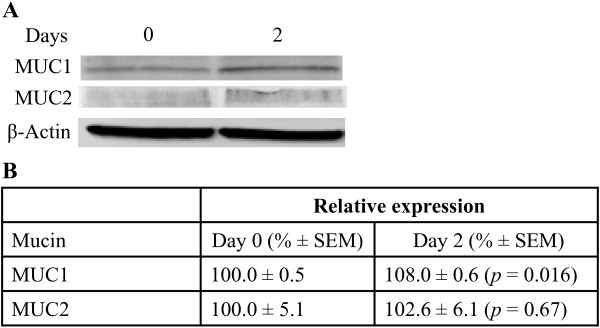
**MUC1 and MUC2 protein expression in LS174T cells detected by Western blot. A:** MUC1, MUC2 and β-actin expression visualised at day 0 and 2; **B:** relative MUC1 and MUC2 chemiluminescence values normalised against β- actin for day 0 and 2. Values associated with means are standard errors of the mean (SEM).

Studies in *C. jejuni* have shown that adherence to and invasion of intestinal epithelial cells is significantly greater when the epithelium is covered in a mucus-like medium of the same viscosity as intestinal mucus, than in medium of zero viscosity [9]. Thus, the decrease in *C. concisus* adherence with increased viscosity differs from that previously observed for *C. jejuni*. To gain a better understanding of the changes in *C. concisus* pathogenesis in viscous environments that more closely resemble the situation within the host, the mucus-producing intestinal goblet-like cell line LS174T [24] was employed. In this experiment we determined if the presence and thickness of the mucus layer affected the attachment and invasion of *C. concisus* UNSWCD. To determine changes in the composition of the mucus layer produced by LS174T cells over time, the levels of MUC1 and MUC2, synthesised by LS174T, we analysed any changes in their expression at two different time points (0 and 2 days). Changes in MUC1 and MUC2 expression were compared relative to day 0 by normalising MUC1 or MUC2 chemiluminescence values against the β-actin control. While MUC1 levels were shown to significantly increase from day 0 to day 2 (*p* = 0.016) (Figure 4A,B), MUC2 expression in LS174T cells did not change from day 0 to day 2 (*p* = 0.67) (Figure 4A,B). Both visually and through the increase in expression of MUC1, we determined that there was a higher accumulation of mucus at the apical surface of LS174T cells at day 2 as compared with day 0.

Given this, we determined using adherence and invasion assays the ability of *C. concisus* UNSWCD to adhere to and invade LS174T cells at day 0 and 2. We observed that the percentage of *C. concisus* UNSWCD adhering to LS174T cells increased from 4.61 ± 0.44% at day 0 to 8.20 ± 0.50% at day 2 (1.8-fold increase, *p* = 0.0036). Interestingly, the percentage of *C. concisus* UNSWCD that invaded LS174T cells also increased 1.8-fold from day 0 to day 2, although overall the levels of invasion were significantly lower than those observed for Caco-2 cells (~50-fold lower). One possible reason for the increased adherence with increase in mucus is the chemoattractive properties of mucins. For example, Hugdahl *et al.* have reported positive chemotaxis by *C. jejuni* towards mucin [25]. Alternatively, *C. concisus* may bind to mucins to help facilitate adhesion to LS174T cells. Interestingly, a study by McAuley *et al.* has shown that *C. jejuni* binds to oligosaccharide ligands on the surface of mucins using adhesins [26]. Collectively, the increase in adherence of *C. concisus* to the surface of LS174T cells may be due to the interplay between the chemoattractive property of mucins along with the ability of *C. concisus* to bind to mucin structures.

While the observed increase in invasion may have resulted from increased adherence, another explanation could be that exposure to mucus also modulates the invasive potential of *C. concisus*. For example, Tu *et al.* have reported that exposure of *C. jejuni* to mucin results in the upregulation of the *Campylobacter* invasion antigen *ciaB* that allows the internalisation of *C. jejuni* into mammalian cells, and that *cadF* which encodes fibronectin binding protein facilitates binding to host fibronectin and allows adhesion to the surface of host cells [27]. These proteins are also encoded by *C. concisus* UNSWCD [28], and whilst studies involving the change in gene expression of *C. concisus* in the presence of mucus have not been conducted, the study in *C. jejuni* raises the possibility that *C. concisus* UNSWCD gene regulation may also occur in the presence of mucus.

Our results with the LS174T cell line are consistent with changes in pathogenic potential of *C. jejuni* in viscous environments, although they do not explain the drop in adherence observed with addition of CMC. One possible reason for this is that unlike CMC, mucus is chemoattractive and bacteria bind to mucins. An alternate explanation is that in the CMC experiments the whole medium (~ 1 cm in height) is viscous, thus, the bacteria are exposed to viscosity directly upon inoculation, whereas in the mucus cell line bacteria are only exposed to the viscous environment (~800 μm in height) upon swimming down to the cells.

### *Biofilm formation by* Campylobacter concisus

**Figure 5:**
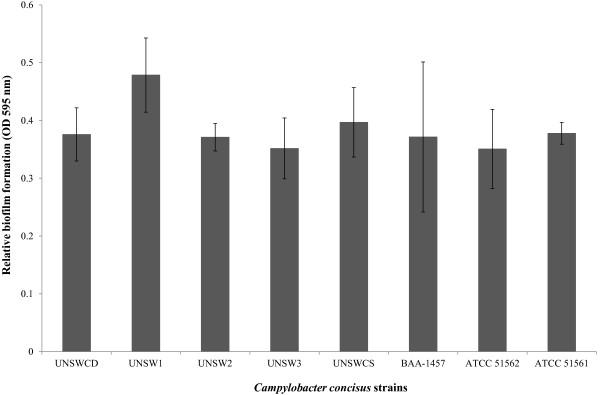
**The ability of *Campylobacter concisus *strains to form biofilms.** The negative control for non-specific binding has been deducted from the OD readings. Vertical lines associated with histogram bars represent standard error of the mean.

Previous studies by our group, which examined the attachment of *C. concisus* strains to Caco-2 cells using ScEM have shown that several *C. concisus* strains adhere in an aggregative pattern on the surface of Caco-2 cells [5,12]. This finding led us to postulate that this aggregation may actually represent biofilm formation. To determine the ability of the eight *C. concisus* strains to form biofilms, the method of Gunther IV *et al.*[16] was optimised. All *C. concisus* strains showed the formation of biofilms (Figure 5). There was no significant difference in the amount of biofilm among the eight strains (F_7,20_ = 0.30, *p* = 0.94).

Given that biofilm formation is reported to assist in bacterial survival, colonisation and protection from host immune responses and antibacterial therapies [29], the ability of *C. concisus* to form biofilms may represent an important virulence mechanism in relation to its pathogenesis and transmission. Interestingly, based on an observation that an increased level of biofilm formation occurs in *C. jejuni* when incubated under aerobic conditions, Reuter *et al.* suggested that biofilms may aid the survival of *C. jejuni* in the environment, and that this adaptation may contribute to its zoonotic lifestyle [30]. While currently it is unknown whether *C. concisus* is a zoonotic infection, the possibility has been raised by the detection of *C. concisus* in cats, dogs, chickens and cattle [3]. In relation to survival within the host, our observation of biofilm like aggregations following infection of intestinal cells with *C. concisus* raises the possibility that like uropathogenic *E. coli* UTI89, biofilm formation may be a critical factor contributing to a persistent infection in the human host [31]. Given that within the intestinal tract *C. concisus* is continually subjected to peristalsis and mucus turn-over, the ability to produce biofilms may allow the bacterium to remain within its niche.

## Conclusions

Evidence suggests that both animals and the oral cavity of humans provide a reservoir of *C. concisus*[3] that could pass into the intestinal tract of humans following ingestion. Based on the results of this study, we hypothesise that strains with higher motility have a greater chance to swim through the intestinal mucus layer and reach the epithelial surface. Once adhered to the epithelium through their flagellum, strains with the proper pathogenicity factors such as the exotoxin 9, which has been associated with the invasive potential of *C. concisus*[5], can invade into the host cell, induce an inflammatory response, and subsequently, cause disease.

## Competing interests

The author’s declare that they have no competing interests.

## Authors’ contribution

PL performed the motility assays, biofilm assays and cell-based assays on Caco-2 cells, analysed the data, and assisted in drafting the manuscript. NOK performed the Western blotting analysis, supervised all experimental work, analysed the data, and drafted the manuscript. KDH performed all statistical analyses. NK performed the cell-based assays on LS174T cells. HMM conceived the study, supervised all experimental work, analysed the data and drafted the manuscript. All authors read and approved the final manuscript.

## Supplementary Material

Additional file 1 **The viscosity of different concentrations of methyl-cellulose.** The data gained from Schneider et al was extrapolated using a polynomial equation. Click here for file

Additional file 2 **The effect of *Campylobacter concisus* concentration on the distance travelled through semi-solid agar.** Click here for file
