# Supplementary material for: Investigation of motility and biofilm formation by intestinal Campylobacter concisus strains
Source: Gut Pathog. 2012 Dec 14;4:22. doi: 10.1186/1757-4749-4-22 (PMC3548772; doi:10.1186/1757-4749-4-22)
Supplement: Additional file 2 — The effect of Campylobacter concisus concentration on the distance travelled through semi-solid agar. [file 1757-4749-4-22-S2.docx]

**Table S1**

**The effect of *Campylobacter concisus* concentration on the distance travelled through semi-solid agar.**

| OD at 595 nm | Cell density (CFU/ml) | Distance (cm) |
| --- | --- | --- |
| 0.920 | 4.0 x 10^9^ | 1.5 |
| 0.495 | 1.9 x 10^9^ | 1.6 |
| 0.346 | 1.2 x 10^9^ | 1.6 |
| 0.271 | 9.6 x 10^8^ | 1.6 |
| 0.178 | 8.1 x 10^8^ | 1.6 |
| 0.089 | 4.8 x 10^8^ | 1.6 |
| 0.079 | 2.9 x 10^8^ | 1.5 |
| 0.054 | 2.5 x 10^8^ | 1.6 |

A preliminary study was conducted in order to determine the optimal concentration of *C. concisus* to inoculate onto semi-solid agar plates for the motility testing studies. This showed that regardless of the concentration (OD) of the *C. concisus* used there was no difference in the distance the bacterium travelled through the agar. Thus, an OD of 0.5 was employed because the zone of motility was most easily detected at that concentration of bacteria.
